# A two-layered brain network model and its chimera state

**DOI:** 10.1038/s41598-019-50969-5

**Published:** 2019-10-07

**Authors:** Ling Kang, Changhai Tian, Siyu Huo, Zonghua Liu

**Affiliations:** 1 Department of Physics, East China Normal University, Shanghai, 200062 P.R. China; 2 School of Data Science, Tongren University, Tongren, 554300 P.R. China

**Keywords:** Statistical physics, thermodynamics and nonlinear dynamics, Complex networks

## Abstract

Based on the data of cerebral cortex, we present a two-layered brain network model of coupled neurons where the two layers represent the left and right hemispheres of cerebral cortex, respectively, and the links between the two layers represent the inter-couplings through the corpus callosum. By this model we show that abundant patterns of synchronization can be observed, especially the chimera state, depending on the parameters of system such as the coupling strengths and coupling phase. Further, we extend the model to a more general two-layered network to better understand the mechanism of the observed patterns, where each hemisphere of cerebral cortex is replaced by a highly clustered subnetwork. We find that the number of inter-couplings is another key parameter for the emergence of chimera states. Thus, the chimera states come from a matching between the structure parameters such as the number of inter-couplings and clustering coefficient etc and the dynamics parameters such as the intra-, inter-coupling strengths and coupling phase etc. A brief theoretical analysis is provided to explain the borderline of synchronization. These findings may provide helpful clues to understand the mechanism of brain functions.

## Introduction

In the fields of nonlinear dynamics and complex network, a long standing and fascinating topic is the understanding of brain functions. Many experiments have shown that the brain dynamics/cortical waves span orders of magnitude in space and time^1,2^, especially in human sleep spindles^3^ and slow-wave sleep^4^, and thus open many questions. For examples, what is the basis for neural population to show rich activities? How do particular activities/waves appear and disperse or do there exist deeper unifying principles? To answer these questions, numerous efforts have been taken and greater progresses have been achieved. For example, previous studies have shown the relationship between the electrical activity of brain and complex psychophysiological processes such as alertness^5^, arousal^6^, attention^7^, memory^8^, and executive functions^9^. Moreover, it has been observed that in brain, functional assemblies of neurons may display distinct interdependent synchronous oscillations^10^, and abnormal synchronization are closely related to epileptic seizure^11,12^.

Many evidences have shown that brain functions are driven by dynamic interactions between large-scale neural circuits or networks^13^, indicating that the underlying anatomical connectivity of the brain provides a crucial backbone to brain functions. It has been hypothesized that cognitive responses and human behavior are the outcome of complex interactions between network structure and regional populations of neurons^14–16^. However, fundamental principles constraining these dynamic network processes have remained elusive. A promising way toward this direction is the partial synchronization, which has been intensively studied in the last decade from two aspects, i.e. chimera state^17–30^ and cluster synchronization^31–43^. The former represents the coexistence of coherent (synchronized) and incoherent (desynchronized) patterns of identical oscillators^20,44^, while the latter denotes the case where the oscillators synchronize with one another in groups, but there is no synchronization among the groups.

Chimera state was first observed by Kuramoto and Battogtokh in 2002^17^ and then named by Abrams and Strogatz in 2004^20^. After that, the study of chimera state has been the focus of extensive research in a wide number of models, including the neuron systems^45–52^, chaotic oscillators^53,54^, high dimensional systems^18,55–58^, and experimental systems^33,59–63^, see reviews^64,65^ for details. For examples, Uhlhaas and Singer pointed out in 2006 that chimera state is strongly connected to various types of neuronal diseases such as Parkinsons disease, epileptic seizures, Alzheimers disease, schizophrenia and brain tumors^66^. Abrams *et al*. constructed a simplest system of chimera state in 2008, which consists of two clusters of *N* identical oscillators^44^. Sethia *et al*. examined the case of time-delay in 2008 and found clustered chimera states^19^. To explain the alternative patterns between the hemispheres over time from the EEG data on dolphins^67^, Ma *et al*. considered a two-cluster network with environmental forcing in 2010 and found that with proper tuning of the interaction strength, the two clusters alternate between coherence and incoherence^68^. Laing showed in 2011 that chimera or chimera-like states have strong connection to the bump behavior of neuronal networks, which has been associated with the mechanisms of visual systems, head direction systems and working memory^69^. In 2013, Omel’chenko demonstrated multiple chimera states in a ring of non-locally coupled phase oscillators^70^. In 2014, Zhu *et al*. discussed chimera state in complex networks^71^. Recently, some attention has been even paid to the control of chimera state^72–76^. Instead of passively observing chimera states, the aim of control is to actively exploit chimeras for applications by making the spatial location accessible. For example, the controlled position of localized synchrony may encode information and perform computations^74^.

Chimera state has been successfully used to explain the phenomenon of *unihemispheric sleep* that during the sleep of some birds and marine mammals, their half brain is synchronized and the other half is unsynchronized^44,68,77–79^. A more interesting example is the *first-night effect* in human sleep^80^, where one hemisphere is more vigilant than the other as a night watch to monitor unfamiliar surroundings during sleep.

While cluster synchronization does not require the coexistence of synchronized and desynchronized groups but only the emergence of synchronized clusters. In cluster synchronization, the local dynamics in synchronized clusters can be different from the dynamics in the other cluster(s)^40,41^. So far, cluster synchronization has been observed in many networked systems, where a network organizes in separate domains of synchronized elements^31–33,35,36,38,39,42^. To understand the underlying mechanism, it has been shown that the formation of clusters is closely related to the symmetries of network topology^34,35,42^. Recently, it was shown that cluster synchronization may also emerge for those nodes that are not related by symmetries but receive the same total amounts of inputs from their neighboring nodes in different clusters^43^. Cluster synchronization has even been experimentally demonstrated in networks^35,40^.

In fact, it is possible for the chimera state and cluster synchronization to show up simultaneously in some systems^32,33^, although they are generally studied independently. A paradigmatic system for this situation is the brain network where neurons and their interconnections through synapses form a very complicated structure. In brain network, neurons are linked together to perform certain tasks and cognitive functions, such as pattern recognition, function approximation, data processing, etc. Recent works have begun to link the existence of chimeras in globally coupled networks to clusters^50,81,82^. However, these works ignore some important factors of real brain network such as the specific structure of the left and right hemispheres and their connection by the corpus callosum. Thus, an intuitive question is what will happen if we consider these factors. To make the problem simple, we here only focus on the study of chimera state but leave the study of cluster synchronization for the next step. In this sense, the question will be equivalent to ask: what will happen if we study the chimera state directly from the data of experimentally measured cerebral cortex, instead of the previous studies on artificial brain networks.

We here answer this question by constructing a two-layered brain network model from the data of cerebral cortex. In this model, we let the two layers represent the left and right hemispheres of cerebral cortex, respectively, and the links between them represent the inter-couplings through the corpus callosum. We focus on how the dynamical patterns of brain network are connected to their structural connectivity, i.e. the key parameters of network, which is also a big issue in the neuroscience. By numerical simulations, we find abundant patterns of chimera states, depending on the parameters of system such as coupling strengths and coupling phase. Further, to provide this finding a solid foundation, we extend the model to a more general two-layered network where each hemisphere of cerebral cortex is replaced by a highly clustered network. We find that the emergence of chimera states depends not only on the structure parameters such as the number of inter-couplings and clustering coefficient etc but also on the dynamics parameters such as the intra-, inter-coupling strengths and coupling phase, i.e. their matching. Very interesting, we find that in phase diagrams, the three states of synchronization, partial synchronization, and disorder are not clearly separated by three regions, but distinguished to each other by many segmented regions, i.e. forming specific boundaries between states. A brief theoretical analysis is provided to explain the borderline of synchronization.

## Results

### A two-layered brain network model based on the data of cerebral cortex

**Figure 1:**
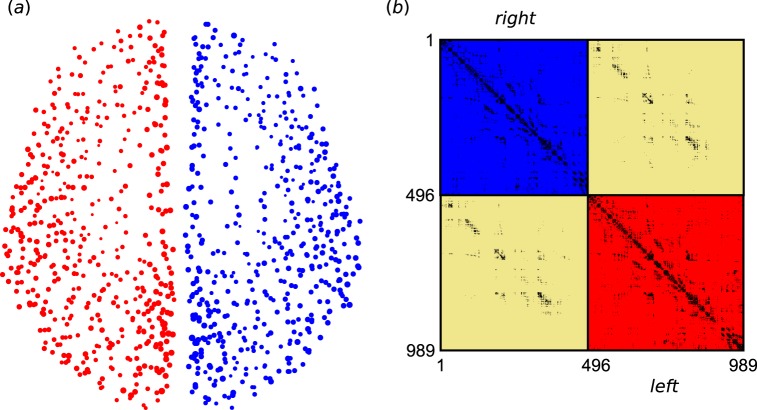
Nodes and links of cerebral cortex from refs^83,84^. (**a**) The 989 nodes distributed on the cerebral cortex. (**b**) Connection matrix of the network of cerebral cortex where the upper left and lower right represent the right and left hemispheres, respectively, and the other two parts are for the inter-connections between the two hemispheres. Each black point denotes a connection.

According to the data measured noninvasively by using diffusion spectrum imaging (DSI) in refs^83,84^, the network of cerebral cortex consists of 998 nodes and 17865 links, where each node represents a cortical region (ROI) and each link between two ROIs is derived from the number of fibers found by the tractography algorithm. By checking the data we find that there are 9 isolated nodes with no links. We remove them in this work, which results in 989 nodes remained. A characteristic feature of this brain network is that it can be divided into two hemispheres connected by the corpus callosum. Figure 1(a) shows the distribution of these 989 nodes on the cerebral cortex, where the subnetwork of right hemisphere has 496 nodes (from *i* = 1 to 496) and the subnetwork of left hemisphere has 493 nodes (from *i* = 497 to 989). We see that they are not homogeneously distributed. Figure 1(b) shows the connection matrix of the 17865 links, where the upper left represents the right hemisphere with 8037 links (i.e. 〈*k*〉 ≈ 32.4), the lower right represents the left hemisphere with 7773 links (i.e. 〈*k*〉 ≈ 31.5), and the other two parts represent the 2055 inter-connected links between the two hemispheres.

**Figure 2:**
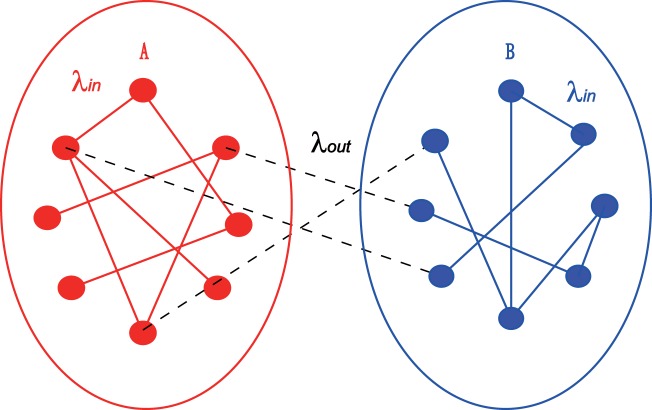
Schematic figure of the two-layered brain network model where *A* and *B* represent the two hemispheres, respectively, “circles” denote the nodes or neurons, and *λ*_*in*_ and *λ*_*out*_ represent the intra- and inter-coupling strengths, respectively.

Intuitively, we can consider Fig. 1 as a two-layered brain network model where the two layers denote the two hemispheres, respectively. The links between *A* and *B* represent the inter-couplings between them through the corpus callosum. Figure 2 is the schematic figure of this model, where the parameter *λ*_*in*_ represents the coupling strength of the links in each of the two-layers and *λ*_*out*_ the coupling strength between the two-layers. To distinguish the intra- and inter-couplings, we let *λ*_*in*_ and *λ*_*out*_ be different. We let ${\ell }_{out}$ be the number of inter-connected links, which equals 2055 in Fig. 1(b).

To conveniently discuss the dynamics of brain network, we here mainly consider the case that both the intra- and inter-couplings are the electric coupling. While the discussion on the case of chemical inter-coupling will be put into the Supplementary Information (SI). Each node of Fig. 2 represents a neuron and the connection between two nodes is implemented by the connection matrix of Fig. 1(b). For convenience, we let the following FitzHugh-Nagumo (FHN) neuron to represent the behavior of each node in the network *A* of Fig. 2

1 $$\begin{array}{rcl}\epsilon {\dot{u}}_{i}^{a} & = & {u}_{i}^{a}-\frac{{({u}_{i}^{a})}^{3}}{3}-{v}_{i}^{a}\\  &  & +\,\frac{{\lambda }_{in}}{{k}_{in,i}^{a}}\mathop{\sum }\limits_{j\mathrm{=1}}^{N}\,{A}_{ij}[{d}_{uu}({u}_{j}^{a}-{u}_{i}^{a})+{d}_{uv}({v}_{j}^{a}-{v}_{i}^{a})]\\  &  & +\,\frac{{\lambda }_{out}}{{k}_{out,i}^{a}}\mathop{\sum }\limits_{j\mathrm{=1}}^{N}\,{(AB)}_{ij}[{d}_{uu}({u}_{j}^{b}-{u}_{i}^{a})+{d}_{uv}({v}_{j}^{b}-{v}_{i}^{a})]\\ {\dot{v}}_{i}^{a} & = & {u}_{i}^{a}+a\\  &  & +\,\frac{{\lambda }_{in}}{{k}_{in,i}^{a}}\,\mathop{\sum }\limits_{j\mathrm{=1}}^{N}\,{A}_{ij}[{d}_{vu}({u}_{j}^{a}-{u}_{i}^{a})+{d}_{vv}({v}_{j}^{a}-{v}_{i}^{a})]\\  &  & +\,\frac{{\lambda }_{out}}{{k}_{out,i}^{a}}\mathop{\sum }\limits_{j\mathrm{=1}}^{N}\,{(AB)}_{ij}[{d}_{vu}({u}_{j}^{b}-{u}_{i}^{a})+{d}_{vv}({v}_{j}^{b}-{v}_{i}^{a})]\end{array}$$

where *i* = 1, 2, …, *N*, *u*_*i*_^*a*^ and *v*_*i*_^*a*^ denote the fast and slow variables, respectively. *k*_*in*,*i*_^*a*^ and *k*_*out*,*i*_^*a*^ are the intra- and inter-degrees of node *i*, respectively. *A*_*ij*_ and (*AB*)_*ij*_ denote the intra- and inter-coupling matrices, respectively. $\epsilon$ is taken as $\epsilon$ = 0.05. *a* is a parameter so that an isolated neuron will be in the excitable state when |*a*| > 1 and oscillatory state when |*a*| < 1^1,45,51,85–87^. Considering that our purpose here is to understand how the brain network structure, especially the corpus callosum, influences the collective behaviors of brain, we would like to choose the oscillatory regime in this work, i.e. *a* = 0.5. By following refs^37,45,46^, the coupling is considered as the rotational coupling matrix

2 $$D=(\begin{array}{cc}{d}_{uu} & {d}_{uv}\\ {d}_{vu} & {d}_{vv}\end{array})=(\begin{array}{cc}\cos \,\alpha  & \sin \,\alpha \\ -\sin \,\alpha  & \cos \,\alpha \end{array}),$$

which depends on the coupling phase *α*. This parameter *α* represents the relative phase difference of interacting oscillators.

Similarly, each node in the network *B* of Fig. 2 satisfies

3 $$\begin{array}{rcl}\epsilon {\dot{u}}_{i}^{b} & = & {u}_{i}^{b}-\frac{{({u}_{i}^{b})}^{3}}{3}-{v}_{i}^{b}\\  &  & +\,\frac{{\lambda }_{in}}{{k}_{in,i}^{b}}\mathop{\sum }\limits_{j\mathrm{=1}}^{N}\,{B}_{ij}[{d}_{uu}({u}_{j}^{b}-{u}_{i}^{b})+{d}_{uv}({v}_{j}^{b}-{v}_{i}^{b})]\\  &  & +\,\frac{{\lambda }_{out}}{{k}_{out,i}^{b}}\mathop{\sum }\limits_{j\mathrm{=1}}^{N}\,{(AB)}_{ij}[{d}_{uu}({u}_{j}^{a}-{u}_{i}^{b})+{d}_{uv}({v}_{j}^{a}-{v}_{i}^{b})]\\ {\dot{v}}_{i}^{b} & = & {u}_{i}^{b}+a\\  &  & +\,\frac{{\lambda }_{in}}{{k}_{in,i}^{b}}\mathop{\sum }\limits_{j\mathrm{=1}}^{N}\,{B}_{ij}[{d}_{vu}({u}_{j}^{b}-{u}_{i}^{b})+{d}_{vv}({v}_{j}^{b}-{v}_{i}^{b})]\\  &  & +\,\frac{{\lambda }_{out}}{{k}_{out,i}^{b}}\mathop{\sum }\limits_{j\mathrm{=1}}^{N}\,{(AB)}_{ij}[{d}_{vu}({u}_{j}^{a}-{u}_{i}^{b})+{d}_{vv}({v}_{j}^{a}-{v}_{i}^{b})]\end{array}$$

We introduce an average phase velocity to study the collective behaviors of Eqs (1) and (3), defined as

4 $${\omega }_{i}^{a}=\frac{2\pi {M}_{i}}{\Delta T}\,i=\mathrm{1,}\,\mathrm{2,}\,\cdots ,\,N$$

for the *i*-th node in the network *A* of Fig. 2, where Δ*T* is the measured time interval in stabilized state and *M*_*i*_ is the measured firing number of node *i* in this time interval, with *M*_*i*_ being large enough. In the same way, we define *ω*_*i*_^*b*^ for the network *B* of Fig. 2. In the following, we study how the parameters *α*, *λ*_*in*_ and *λ*_*out*_ influence the collective behaviors of Eqs (1) and (3).

**Figure 3:**
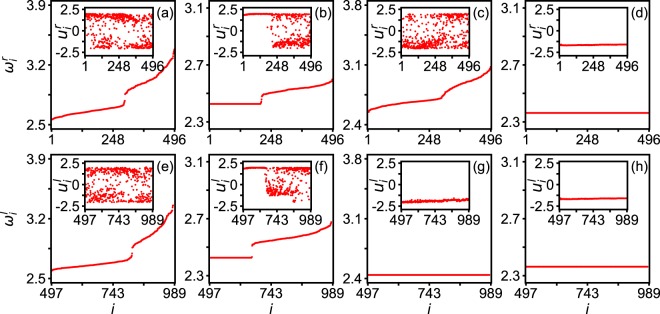
Four typical behaviors in the cerebral cortex of Fig. 1 with *α* = *π*/2 − 0.1 where the up and down panels represent the two hemispheres, respectively, and the insets are their corresponding dynamics of *u*_*i*_ at a moment *t*. The parameters are *λ*_*in*_ = 0.1 and *λ*_*out*_ = 0.3 in panels (**a**,**e**) of disorder; *λ*_*in*_ = 0.1 and *λ*_*out*_ = 1.8 in panels (**b**,**f**) of chimera state; *λ*_*in*_ = 0.4 and *λ*_*out*_ = 3.5 in panels (**c**,**g**) of an emergent state conceptually similar to the state of unihemispheric sleep; and *λ*_*in*_ = 4.0 and *λ*_*out*_ = 3.5 in panels (**d**,**h**) of synchronization.

As the concept of the space is lost on complex networks, we here follow the ref.^71^ to rearrange the number of oscillators by the ascending order of *ω*_*i*_ such that *i* ≥ *j* if *ω*_*i*_ ≥ *ω*_*j*_. Then, we integrate Eqs (1) and (3) by randomly choosing the initial conditions of *u*_*i*_(0) and *v*_*i*_(0) as in previous studies^28,45,52,88^. But we find that the observed results can be also obtained by other initial conditions, i.e. robust to initial conditions. Figure 3 shows the stabilized results of rearranged *ω*_*i*_ for four typical cases where the up panels are for the network-A, down panels for the network-B, and the insets are their corresponding dynamics of *u*_*i*_ at a moment *t*. The parameter *α* is fixed as *α* = *π*/2 − 0.1. The panels (a) and (e) represent a typical case of disorder with *λ*_*in*_ = 0.1 and *λ*_*out*_ = 0.3; (b) and (f) a typical case of chimera state with *λ*_*in*_ = 0.1 and *λ*_*out*_ = 1.8; (c) and (g) a specific case of disordered network-A and synchronized network-B, i.e. an emergent state conceptually similar to the state of unihemispheric sleep, with *λ*_*in*_ = 0.4 and *λ*_*out*_ = 3.5; and (d) and (h) a typical case of synchronization with *λ*_*in*_ = 4.0 and *λ*_*out*_ = 3.5. We see that there is a plateau of *ω*_*i*_ for the case of chimera state, a constant of *ω*_*i*_ for the case of synchronization, and randomly distributed *ω*_*i*_ for the case of disorder. From the insets of Fig. 3(b,f) we see that the synchronized and unsynchronized *u*_*i*_(*t*) are coexistent, marking the feature of chimera state. And from the insets of Fig. 3(c,g) we see that *u*_*i*_(*t*) is disordered in one hemisphere but synchronized in another one, marking the first-night effect. Our numerical simulations further show that the parameter region for the unihemispheric sleep-like emergent state is much smaller than that of chimera state, confirming the difficulty of observing the first-night effect.

**Figure 4:**
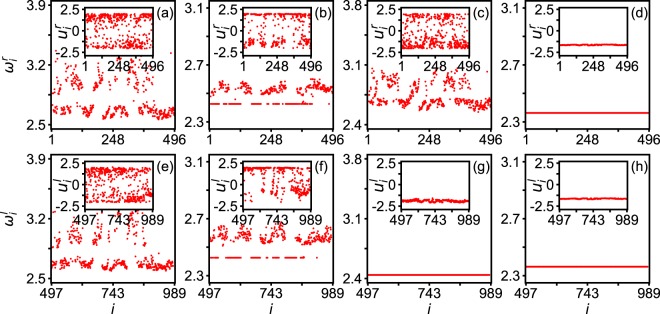
Corresponding case of Fig. 3 without rearranging the order of oscillators where all the parameters in each panel are the same as in the corresponding panels of Fig. 3.

It will be interesting to see what will happen if we do not rearrange the order of oscillators. Figure 4 shows the results corresponding to Fig. 3, without rearranging the order of oscillators. From Fig. 4(a,e) we see that both *ω*_*i*_^*a*^ and *ω*_*i*_^*b*^ are randomly distributed, which are consistent with the varying *ω*_*i*_^*a*^ and *ω*_*i*_^*b*^ in Fig. 3(a,e). From Fig. 4(b,f) we see that both *ω*_*i*_^*a*^ and *ω*_*i*_^*b*^ are divided into two parts, i.e. one constant and the other non-constant, which are consistent with the constant and varying parts of *ω*_*i*_^*a*^ and *ω*_*i*_^*b*^ in Fig. 3(b,f). From Fig. 4(c,g) we see that *ω*_*i*_^*a*^ are disordered while *ω*_*i*_^*b*^ are synchronized, which are consistent with the varying *ω*_*i*_^*a*^ and constant *ω*_*i*_^*b*^ in Fig. 3(c,g). Further, we see that Fig. 4(d,h) are the same as Fig. 3(d,h). Thus, we conclude that Fig. 3 reflects the same phenomena as that of Fig. 4, except that it is much easier to distinguish the chimera state in Fig. 3 than in Fig. 4. In the following, we will use the approach of rearranging the order of oscillators.

On the other hand, it is maybe necessary to discuss the neuroscientific inferences of parameter ranges. For this purpose, we pay special attention to the coupling strengths of *λ*_*in*_ and *λ*_*out*_ in the different states of Figs 3 and 4. We may notice that both *λ*_*in*_ and *λ*_*out*_ are weak in the disorder state of Figs 3(a,e) and 4(a,e); *λ*_*in*_ is weak but *λ*_*out*_ is relatively strong in the chimera state of Figs 3(b,f) and 4(b,f); *λ*_*in*_ is relatively strong but *λ*_*out*_ is very strong in the unihemispheric sleep-like state of Figs 3(c,g) and 4(c,g); and both *λ*_*in*_ and *λ*_*out*_ are very strong in the synchronization state of Figs 3(d,h) and 4(d,h). These values tell us some neuroscientific information. Firstly, weak *λ*_*in*_ and *λ*_*out*_ imply that both the intra- and inter-couplings are not strong, indicating no running brain functions. A typical situation of this state is the resting state of brain with random behaviors. Secondly, for the case of weak *λ*_*in*_ and relatively strong *λ*_*out*_, the relatively strong *λ*_*out*_ implies some communications between the two hemispheres of cerebral cortex, indicating a normal functional state involved an ensemble of neurons distributed in different brain regions. Thirdly, the case of relatively strong *λ*_*in*_ but very strong *λ*_*out*_ is beyond the couplings for a normal brain functional state, which may be launched by a vigilance and thus is consistent with the case of the first-night effect. Finally, the case of both very strong *λ*_*in*_ and *λ*_*out*_ represents an abnormal synchronization, which is well known for epileptic seizures.

From Fig. 3 we see that the chimera state may appear, provided that the parameters *α*, *λ*_*in*_, and *λ*_*out*_ are matched. We wonder whether it is possible to observe chimera state for other values of these parameters. For this purpose, we study how the parameters of both network structure and dynamics influence the emergence of chimera state in the whole parameter plane. We would like to take the measure of chimera state in ref.^89^ introduced by Kemeth *et al*. for a general classification of chimera patterns. In their approach, a measure *g*_0_ is used to characterize the degree of spatial coherence, with *g*_0_ = 1 for a fully synchronization, *g*_0_ ≈ 0 for incoherence, and 0 < *g*_0_ < 1 for chimera patterns. In this work, we use its average *g*_1_ = 〈*g*_0_(*t*)〉_*t*_ to measure the degree of synchronization, i.e. *g*_1_ = 1 for a fully synchronization, *g*_1_ ≈ 0 for incoherence, and 0 < *g*_1_ < 1 for chimera patterns.

In ref.^89^, the state with 0 < *g*_1_ < 1 is defined as chimera state. Using this approach to the case of cluster synchronization, we will have *g*_1_ ≈ 1 when the system can be divided into a few synchronized clusters. In this case, the value of *g*_1_ can be used to distinguish the chimera state and cluster synchronization, but it cannot distinguish the fully synchronization and cluster synchronization. However, when the number of synchronized clusters in a network is very large, the boundary oscillators between synchronized clusters will be also large and thus have a substantial contribution to *g*_1_. As these boundary oscillators represent the spatial heterogeneity and thus contribute a local *g*_1_ = 0, resulting in a 0 < *g*_1_ < 1 for the whole system. In this case, the value of *g*_1_ cannot be used to distinguish the chimera state and cluster synchronization. To avoid confusion, we here call the state with 0 < *g*_1_ < 1 as partial synchronization, without distinguishing whether it is the chimera state or cluster synchronization. Additionally, the system will be chimera state when their snapshots of *u*_*i*_ or *v*_*i*_ show a coexistence of coherence and incoherence.

**Figure 5:**
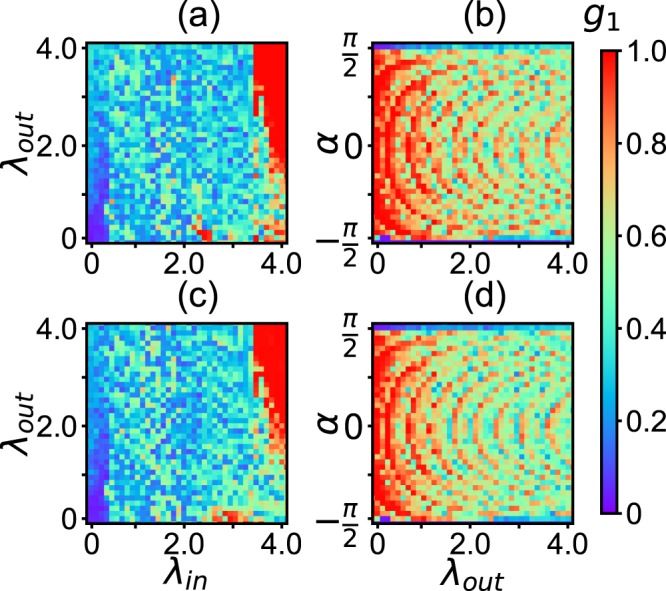
Phase diagram of *g*_1_ for the network of cerebral cortex with electric inter-coupling, where the up panels are for the right hemisphere and down panels for the left hemisphere. (**a**,**c**) represent the values of *g*_1_ in the parameter plane of *λ*_*in*_ and *λ*_*out*_ for *α* = *π*/2 − 0.1, and (**b**,**d**) the case in the parameter plane of *λ*_*out*_ and *α* for fixed *λ*_*in*_ = 3.0.

We now study how the parameters of both network and dynamics influence the collective behaviours of system. Figure 5 shows the results where the up and down panels are for the two networks-A and -B, respectively, (a) and (c) represent the values of *g*_1_ in the parameter plane of *λ*_*in*_ and *λ*_*out*_ for fixed *α* = *π*/2 − 0.1, and (b) and (d) the case in the parameter plane of *λ*_*out*_ and *α* for fixed *λ*_*in*_ = 3.0. From the four panels of Fig. 5 we see that the three states of disorder, partial synchronization and synchronization are distributed in the phase diagram and their individual regions are not very large, indicating that all the parameters *α*, *λ*_*in*_, and *λ*_*out*_ are the key factors to cooperate to make the collective behaviors.

### Extension of the two-layered model to a general case of human brain network

Noticing that the network of Fig. 1 is only for a specific brain network, it is necessary to extend it to a general case of human brain network, within the framework of Fig. 2. For this purpose, we keep the characteristic features of Fig. 1 but allow the key parameters such as the size *N*, the coupling strengths *λ*_*in*_ and *λ*_*out*_, and the number of inter-coupling links ${\ell }_{out}$ to be changeable.

**Figure 6:**
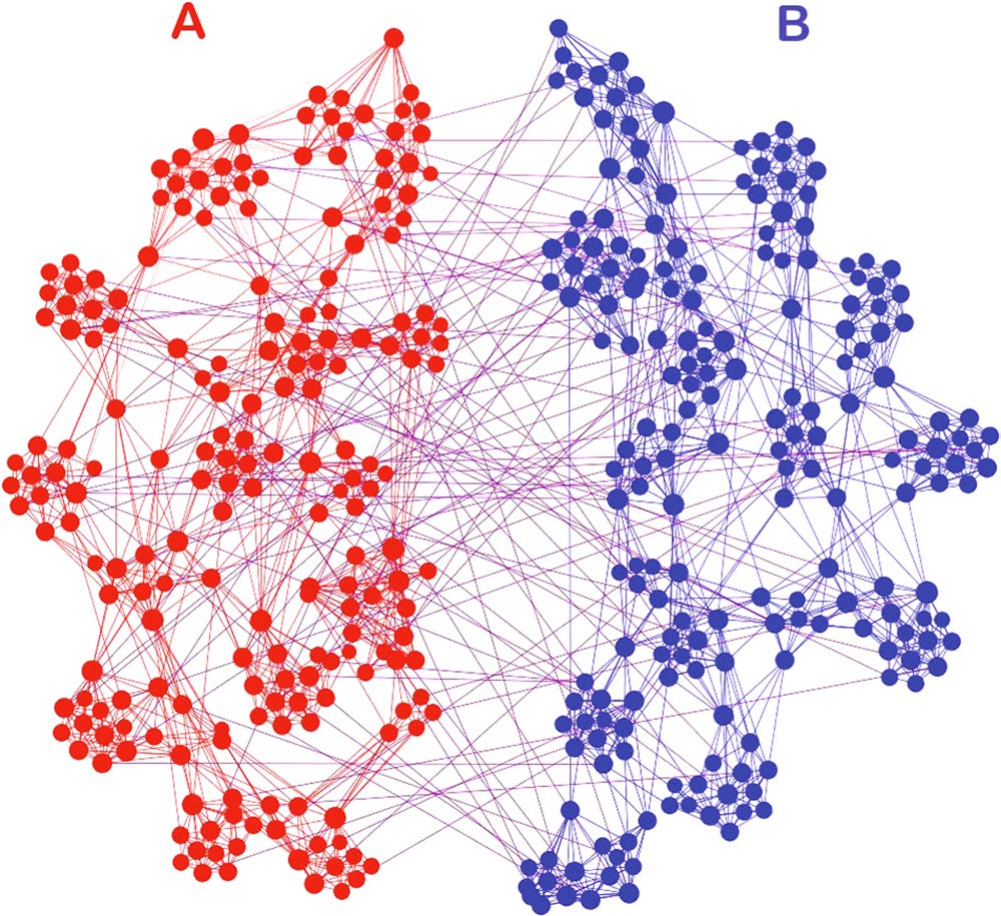
A general model of brain network with size *N* = 200, average degree 〈*k*〉 = 10, and clustering coefficient *C* = 0.7 in each subnetwork, which is rewired from a random network by the algorithm of the rewiring approach^91^.

It is well known that the brain network has a small-world topology characterized by dense local clustering and a short path length between any (distant) pair of nodes due to the existence of relatively few long-range connections^4,90^. This modular organization can support both segregated/specialized and distributed/integrated information processing. In this sense, a general model of brain network has to be a modular network, represented by a larger clustering coefficient *C*. We here use the algorithm of the rewiring approach^91^ to generate this modular network from a random network. In detail, we first start from two random subnetworks with size *N* = 200 and the average degree 〈*k*〉 = 10, i.e. the total size of network is 2*N* = 400. We gradually increase their clustering coefficient *C* to a larger value. Then, we randomly add links between the two subnetworks A and B until the number of inter-connected links reaches ${\ell }_{out}$. Figure 6 shows the obtained network with *C* = 0.7, which will be considered as the general model of brain network in this paper.

**Figure 7:**
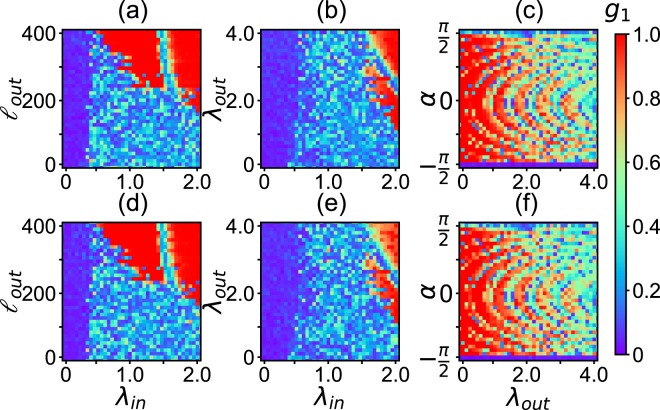
Phase diagram of *g*_1_ with *N* = 200 and 〈*k*〉 = 10, where the up panels are for the network-A and down panels for the network-B. (**a**,**d**) represent the values of *g*_1_ in the parameter plane of *λ*_*in*_ and ${\ell }_{out}$ for fixed *λ*_*out*_ = 0.5 and *α* = *π*/2 − 0.1, (**b**,**e**) the case in the parameter plane of *λ*_*in*_ and *λ*_*out*_ for fixed ${\ell }_{out}\mathrm{=100}$ and *α* = *π*/2 − 0.1, and (**c**,**f**) the case in the parameter plane of *λ*_*out*_ and *α* for fixed ${\ell }_{out}\mathrm{=100}$ and *λ*_*in*_ = 0.6.

Now, we study the influence of the key parameters *λ*_*in*_, *λ*_*out*_ and ${\ell }_{out}$ on the dynamics of the network in Fig. 6. For this purpose, we also let the nodes of Fig. 6(a,b) be represented by the Eqs (1) and (3), respectively. We interestingly find that the general model of Fig. 6 can show the similar behaviors as in Fig. 5 such as the collective behaviors of synchronization, partial synchronization, and disorder, depending on the parameters *λ*_*in*_, *λ*_*out*_, ${\ell }_{out}$ and *α*. Figure 7 shows the results.

From Fig. 7(a,d) we see that the number of inter-coupling links ${\ell }_{out}$ does not take effect when *λ*_*in*_ is small but take effect when *λ*_*in*_ is large. For the latter, we see that it will be most probably the partial synchronization when ${\ell }_{out}$ is less than 200 but synchronization when ${\ell }_{out}$ is larger than 200. These results can be understood as follows. When *λ*_*in*_ < 0.5, the intra-coupling is too weak to induce a collective behavior. In this situation, only a very large inter-coupling or a larger ${\ell }_{out}$ can compensate the intra-coupling to make a partial synchronization or synchronization. However, when *λ*_*in*_ > 0.5, the intra-coupling is large enough to induce a partial synchronization. In this situation, the inter-coupling ${\ell }_{out}$ can help to induce synchronization when ${\ell }_{out}$ is larger than 200.

**Figure 8:**
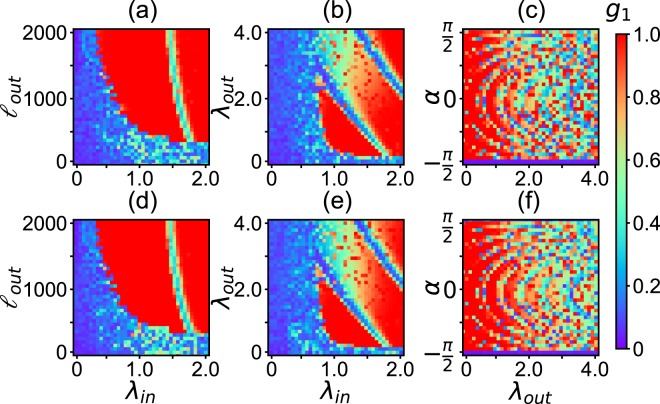
Phase diagram of *g*_1_ with *N* = 1000 and 〈*k*〉 = 50, where the up panels are for the network-A and down panels for the network-B. (**a**,**d**) represent the values of *g*_1_ in the parameter plane of *λ*_*in*_ and ${\ell }_{out}$ for fixed *λ*_*out*_ = 0.5 and *α* = *π*/2 − 0.1, (**b**,**e**) the case in the parameter plane of *λ*_*in*_ and *λ*_*out*_ for fixed ${\ell }_{out}\mathrm{=500}$ and *α* = *π*/2 − 0.1, and (**c**,**f**) the case in the parameter plane of *λ*_*out*_ and *α* for fixed ${\ell }_{out}\mathrm{=500}$ and *λ*_*in*_ = 0.8.

Considering that the real cerebral cortex of Fig. 1 has a network size much larger than *N* = 200, it is necessary to discuss the robustness of size *N* in the general model of human brain network. For this purpose, we here consider a case of Fig. 6 with *N* = 1000, average degree 〈*k*〉 = 50, and *C* = 0.7. Figure 8 shows the results where the up panels are for the network-A and down panels for the network-B. Comparing the corresponding panels between Fig. 8 with *N* = 1000 and Fig. 7 with *N* = 200, respectively, we see that they are qualitatively similar to each other, confirming that the collective behaviors are robust to the network size.

### A brief theoretical analysis for the borderline of synchronization

From all the three phase diagrams of Figs 5, 7 and 8 we see that their synchronized regions (the red parts) are divided into different areas. Especially, in the parameter plane of *λ*_*out*_ and *α*, the synchronized areas show arc-shaped patterns. To understand its mechanism, we make a brief theoretical analysis.

In a synchronized state, we have *δ*_*u*_ ≡ *u*_*j*_ − *u*_*i*_ = 0 and *δ*_*v*_ ≡ *v*_*j*_ − *v*_*i*_ = 0. While in an unsynchronized state, both *δ*_*u*_ and *δ*_*v*_ will evolve with time *t*. The borderlines of synchronized regions are the boundaries between synchronization and un-synchronization, thus the values of *δ*_*u*_ and *δ*_*v*_ for those points at the borderlines will be in between the two limits. In this sense, we may approximately assume that both *δ*_*u*_ and *δ*_*v*_ are non-zero and non-time dependent at the borderline of synchronization, i.e. non-zero constants. On the other hand, the total coupling from Eq. (1) can be approximately written as

5 $$y=({\lambda }_{in}+{\lambda }_{out})[\,\cos (\alpha )\delta u+\,\sin (\alpha )\delta v]$$

**Figure 9:**
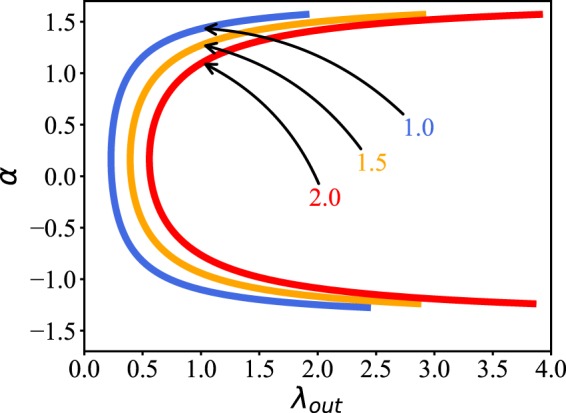
The borderlines of synchronization in the parameter plane of *λ*_*out*_ and *α*, where the parameters are taken as *δu* = 3.0, *δv* = 0.5, *λ*_*in*_ = 0.1, and *α* ∈ (−*π*/2, *π*/2). The three curves correspond to *y* = 1.0, 1.5 and 2.0, respectively.

The behavior of Eq. (1) will be determined by the value of *y*. As all the points on a boundary line have the same state, they will have the same value of *y*, i.e. *y* will be a constant at the borderline of synchronization. Thus, for a given *y*, we may obtain the relationship between *λ*_*out*_ and *α* for the borderline of synchronization by fixing the other variables of Eq. (5). For different *y*, we will have different borderlines of synchronization. Figure 9 shows the results for three typical *y*. We see that all the three curves are arc-shaped, confirming the arc-shaped patterns in the parameter plane of *λ*_*out*_ and *α* of Figs 5, 7 and 8.

This explanation can be also used to explain the relationship between *λ*_*in*_ and *λ*_*out*_ in Figs 7 and 8. By Eq. (5) we have

6 $${\lambda }_{out}=\frac{y}{\cos (\alpha )\delta u+\,\sin (\alpha )\delta v}-{\lambda }_{in}$$

We see that *λ*_*out*_ will linearly decrease with the increase of *λ*_*in*_, which is consistent with the borderlines of synchronization in Figs 7(b,e) and 8(b,e).

## Discussion

### Influence of chemical inter-coupling

The above results are based on the electric coupling, which is the major coupling in brain network. However, there is about 20% chemical coupling in the real brain network. Hence, the interaction between neurons happens through two different synapses, namely, the electrical gap junction and chemical synapses^92^. In general, we have an effect of time delay in the interaction of neurons, due to the limited speed of signal transmission and processing in brain network. For example, the axonal conduction delays depend on the distance between neurons in the brain and can reach up to tens of milliseconds^93^. Time delays comparable to timescales of neuronal oscillations are known to have significant effects in the collective (ensemble) activity of neurons^93–95^. Especially, it is well known that the length of corpus callosum is much longer than the average distance between two neighboring neurons in each of the two hemispheres. To reflect this feature, we may distinguish the intra- and inter-couplings. That is, we ignore the time-delay for all the intra-couplings but consider the influence of time-delay for the inter-couplings. In details, we let all the inter-couplings be the chemical coupling but remain all the intra-couplings be the electric coupling. We find that the chemical inter-coupling, no matter with or without time-delay, will influence the possibility for the brain network to show chimera state. The detailed results are shown in SI.

### Implication of segmented synchronized region in the context of brain

It is well known that the brain operates near a critical point so that it has a rich metastability to sustain stimulus-selective persistent activity for working memory^96–99^. This metastability provides a useful strategy for brain functioning, coding and memory^100–102^. So far, theoretical understanding of brain functions remains very primary, but our results may show some new insights. For example, if we assume that each local area of 0 < *g*_1_ < 1 surrounded by the segmented synchronized regions in the phase diagrams of Figs 7 and 8 corresponds to a specific brain function, the large number of these functional local areas may be considered as the guarantee for the diversity of brain functions. Further, for an arbitrary point in a local area of 0 < *g*_1_ < 1, its value may come from different ways. Take the 66-region parcellation^83,84^ as an example. For a specific brain function, one or a few of the 66-regions will be in synchronized state with *g*_1_ = 1 while the others remain unsynchronized with *g*_1_ = 0, resulting an averaged value of *g*_1_ with 0 < *g*_1_ < 1. This averaged value of *g*_1_ may not change when the synchronized regions are different. Thus, different ways to obtain the same *g*_1_ will also enhance the diversity of brain functions.

### Limitations of the brain network model of Fig. 2

Although the model of Fig. 2 can explain the influence of the key parameters (*λ*_*in*_, *λ*_*out*_, *α* and ${\ell }_{out}$ etc) to the patterns of brain network, we have to point out its limitations. For example, brain network models can be represented more appropriately by weighted connectivity and by including a stochastic element^16^, but we here use neither of these in the model of cerebral cortex. Another limitation is that for simplicity, we use single neuron model to represent the dynamics of a node, i.e. a ROI. This can be better replaced by the mean-field models such as the Wilson-Cowan nonlinear oscillator^103,104^ or the neural mass model^105,106^.

In conclusions, we have presented a two-layered brain network model of coupled neurons to study the collective patterns of brain network, based on the data of cerebral cortex. By this model we find that the two-layered brain network may have different states such as chimera states in either one hemisphere or the whole network, confirming both the unihemispheric sleep for some birds and marine mammals and the first-night effect for human beings. By studying the influence of the key parameters ${\ell }_{out}$, *λ*_*in*_ and *λ*_*out*_, we show that the collective patterns is in fact the result of the matching among them, indicating that different matching will induce different patterns and thus guarantee the diversity of patterns. We also find that the synchronized region in phase diagram is divided into unconnected areas, which has been explained by a brief theoretical analysis.

## Supplementary information

Supplementary information for: A two-layered brain network model and its chimera state
