# Supplementary material for: A two-layered brain network model and its chimera state
Source: Sci Rep. 2019 Oct 7;9:14389. doi: 10.1038/s41598-019-50969-5 (PMC6779761; doi:10.1038/s41598-019-50969-5)
Supplement: Supplementary file 1 — Supplementary information for: A two-layered brain network model and its chimera state [file 41598_2019_50969_MOESM1_ESM.pdf]

Supplementary information for: A two-layered brain network model and its chimera state

Ling Kang,[1] Changhai Tian,[1,2] Siyu Huo,[1] and Zonghua Liu[1]
[1]Department of Physics, East China Normal University, Shanghai, 200062, P. R. China
[2]School of Data Science, Tongren University, Tongren 554300, P. R. China

I. THE CASE OF THE REAL NETWORK OF CEREBRAL CORTEX WITH CHEMICAL INTER-COUPLING

As the main text has discussed the case of electric inter-coupling, we here discuss the case of chemical inter-coupling. Taking the same procedures as in the main text, we first discuss the case of the real network of cerebral cortex and then discuss the general model of brain network in the next section.

In human brain, the communication between the left and right hemispheres has to go through the long corpus callosum and thus causes some time-delay, due to the limited speed of signal transmission and processing. However, the model in the main text does not consider this feature as its inter-coupling is the electric coupling with no time-delay. In general, the electric coupling is a good approximation when the coupling distance is short. But for a long distance coupling, it is better to consider the effect of time-delay. To make the model of the main text to reflect this effect of time-delay along the corpus callosum, we here pay attention to the influence of time-delay on the emergence of chimera state. For convenience, we keep the electric coupling for all the intra-links and let the coupling of inter-links be chemical coupling. Further, we make a simple approximation of uniform delay for all the ℓ_{out} inter-couplings, where the delay is to be interpreted as a mean effective delay. Thus, for the case of chemical inter-coupling, the dynamics of the layer-A will become

$$\begin{aligned} \epsilon\dot{u}_i^a &= u_i^a - \frac{(u_i^a)^3}{3} - v_i^a \qquad (1)\\ &+\frac{\lambda_{in}}{k_{in,i}^a}\sum_{j=1}^{N}A_{ij}[d_{uu}(u_j^a-u_i^a)+d_{uv}(v_j^a-v_i^a)]\\ &+\frac{\lambda_{out}}{k_{out,i}^a}\sum_{j=1}^{N}(AB)_{ij}g_j(u_{th}-u_i^b)\\ \dot{v}_i^a &= u_i^a + a\\ &+\frac{\lambda_{in}}{k_{in,i}^a}\sum_{j=1}^{N}A_{ij}[d_{vu}(u_j^a-u_i^a)+d_{vv}(v_j^a-v_i^a)] \end{aligned}$$

and the dynamics of the layer-B will become

$$\begin{aligned} \epsilon\dot{u}_i^b &= u_i^b - \frac{(u_i^b)^3}{3} - v_i^b \qquad (2)\\ &+\frac{\lambda_{in}}{k_{in,i}^b}\sum_{j=1}^{N}B_{ij}[d_{uu}(u_j^b-u_i^b)+d_{uv}(v_j^b-v_i^b)]\\ &+\frac{\lambda_{out}}{k_{out,i}^b}\sum_{j=1}^{N}(AB)_{ij}g_j(u_{th}-u_i^a)\\ \dot{v}_i^b &= u_i^b + a\\ &+\frac{\lambda_{in}}{k_{in,i}^b}\sum_{j=1}^{N}B_{ij}[d_{vu}(u_j^b-u_i^b)+d_{vv}(v_j^b-v_i^b)] \end{aligned}$$

with

$$g_j = g_{max}[e^{-(t-t_j^s-\tau)/\tau_d} - e^{-(t-t_j^s-\tau)/\tau_r}] \qquad (3)$$

where g_{max} describes the maximal synaptic conductance between neurons, u_{th} denotes the synaptic reversal potential, τ is the time delay between connected neurons, t_j^s represents the presynaptic spiking, τ_d and τ_r stand for the decay and rise time of the function and determine the duration of the response [1–5]. We here take the parameters as $g_{max} = 0.35, u_{th} = 0, \tau_d = 10$, and $\tau_r = 1$.

The inter-coupling in Eqs. (1) and (2) will be a purely chemical coupling when τ equals zero. From Eq. (3) we notice that the synaptic conductance g_j depends on a series of firing times t_j^s, implying that the effect of time-delay is somehow involved even when $\tau = 0$. It is necessary to point out that this effect of time-delay from t_j^s is caused by the temporal aspect of firings, in contrast to the delay τ from spatial distance in Eq. (3). Thus, the chemical inter-coupling in Eqs. (1) and (2) is essentially different from the electric inter-coupling, even in the extreme case of $\tau = 0$. To observe the influence of the purely chemical inter-coupling on chimera state, we first discuss the case of zero time-delay, i.e. $\tau = 0$ in Eq. (3).

Fig. 1 shows the results from Eqs. (1) and (2) with $\tau = 0$ for the real network of cerebral cortex in Fig. 1 of main text. Comparing the corresponding panels between Fig. 1 and Fig. 5 in the main text, we firstly see that the areas of synchronization (the red parts) in Fig. 5 of main text disappear in Fig. 1, indicating that the chemical inter-coupling destroys the global synchronization. This is reasonable as a healthy person usually does not show the behaviour of epileptic seizure characterized by the global synchronization. Secondly, we see that the areas of disorder in Fig. 1 is much larger than that in Fig. 5 of main text, indicating that the conditions for chimera state in real brain network are more rigorous.

Then, we discuss the case of chemical inter-coupling with time-delay. That is, we consider the network of cerebral cor-

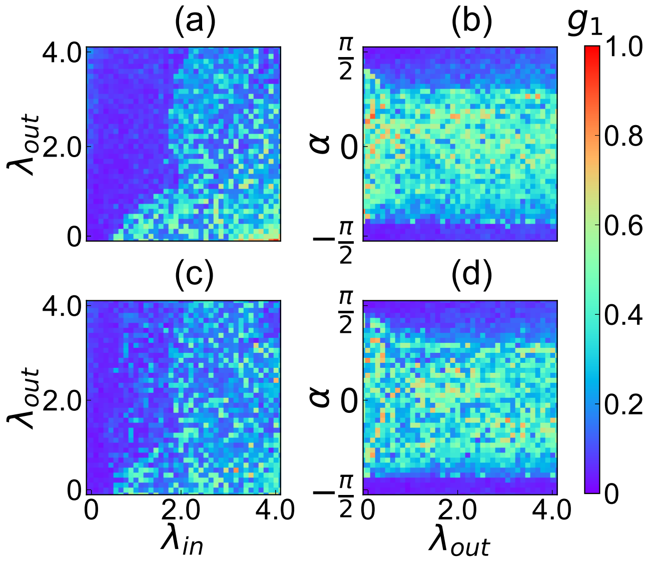

FIG. 1: (color online). Phase diagram of g_1 for the network of cerebral cortex with chemical inter-coupling of $\tau = 0$, where the up and down panels are for the right and left hemispheres, respectively. (a) and (c) represent the values of g_1 in the parameter plane of λ_{in} and λ_{out} for $\alpha = \pi/2 - 0.1$, and (b) and (d) the case in the parameter plane of λ_{out} and α for fixed $\lambda_{in} = 3.0$.

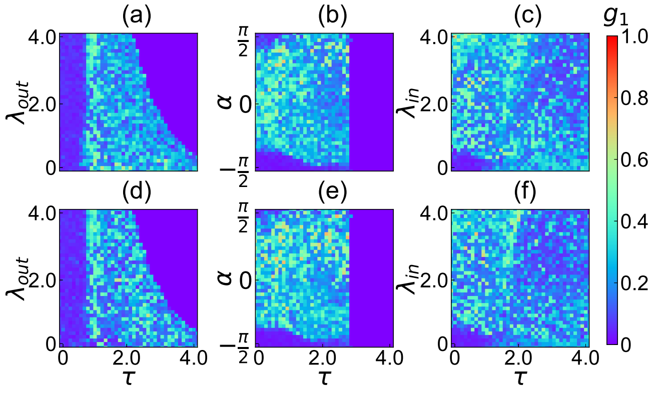

FIG. 2: (color online). Phase diagram of g_1 for the network of cerebral cortex with chemical inter-coupling of $\tau > 0$, where the up and down panels are for the right and left hemispheres, respectively. (a) and (d) represent the values of g_1 in the parameter plane of τ and λ_{out} for fixed $\lambda_{in} = 0.5$ and $\alpha = \pi/2 - 0.1$, (b) and (e) the case in the parameter plane of τ and α for fixed $\lambda_{in} = 0.5$ and $\lambda_{out} = 2.0$, and (c) and (f) the case in the parameter plane of τ and λ_{in} for fixed $\lambda_{out} = 0.5$ and $\alpha = \pi/2 - 0.1$.

tex and the neurons of Eqs. (1) and (2) with $\tau > 0$. Fig. 2 shows the results where the individual panels represent the match between τ and the parameters λ_{out}, α and λ_{in}, respectively. From the panels of Fig. 2(a)-(b) and (d)-(e) we see that except no synchronization as in Fig. 1, there are clearly boundaries between the chimera state and disorder. This property is important for the brain functions. From the panels of Fig. 2(c) and (f) we see that for a larger τ, chimera state can be observed even if λ_{in} is very small, confirming the importance of communication through the corpus callosum.

In sum, the real brain network has a special topology of community structure selected by nature. This structure is favorable not only to the chimera state but also to the distinction between disorder and chimera state.

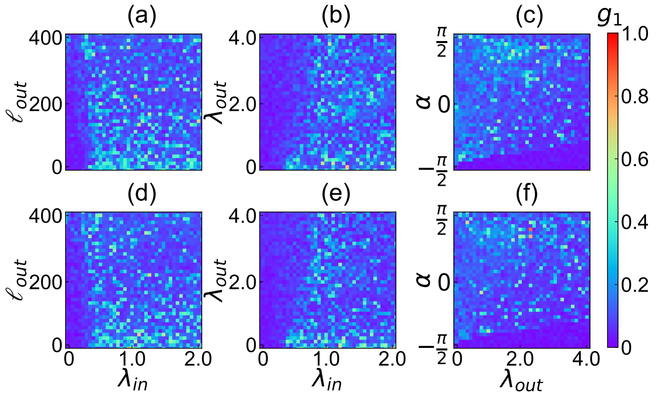

FIG. 3: (color online). Phase diagram of g_1 for the general two-layered brain network model with chemical inter-coupling of $\tau = 0$, where $N = 200, C = 0.7$, $\langle k \rangle = 10$, and the up panels are for the network-A and down panels for the network-B. (a) and (d) represent the values of g_1 in the parameter plane of λ_{in} and ℓ_{out} for fixed $\lambda_{out} = 0.5$ and $\alpha = \pi/2 - 0.1$, (b) and (e) the case in the parameter plane of λ_{in} and λ_{out} for fixed $\ell_{out} = 100$ and $\alpha = \pi/2 - 0.1$, and (c) and (f) the case in the parameter plane of λ_{out} and α for fixed $\ell_{out} = 100$ and $\lambda_{in} = 0.6$.

II. THE CASE OF THE GENERAL TWO-LAYERED BRAIN NETWORK MODEL WITH CHEMICAL INTER-COUPLING

According to the general two-layered brain network model in the main text, we now discuss the case with chemical inter-coupling. In this case, both the network size N and the number of inter-coupling links ℓ_{out} are changeable, in contrast to the fixed N and ℓ_{out} in the above section.

We first discuss the case of $N = 200$ and zero time-delay, i.e. $\tau = 0$ in Eq. (3). Fig. 3 shows the results. Comparing the corresponding panels between Fig. 3 and Fig. 1, we see that they are similar to each other, confirming that the general two-layered brain network model is equivalent to the real network of cerebral cortex.

For the same reason as in the case of electric inter-coupling, we also consider a case of larger network with $N = 1000, C = 0.7$ and $\langle k \rangle = 50$. Fig. 4 shows the results where the up panels are for the network-A and down panels for the network-B. Comparing the corresponding panels between Fig. 4 and Fig. 3, respectively, we find that the chimera states in Fig. 4 is more evident than that of Fig. 3, indicating that a larger network supports more capacity of network patterns.

Then, we move to the case of nonzero time-delay in Eqs. (1) and (2), i.e. with $\tau > 0$ in Eq. (3), and pay attention

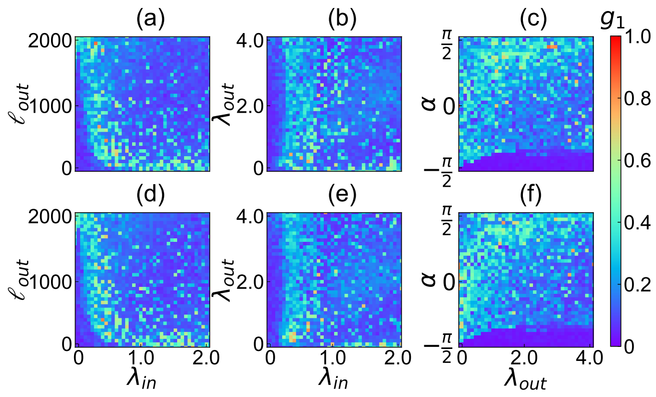

FIG. 4: (color online). Phase diagram of g_1 for the general two-layered brain network model with chemical inter-coupling of $\tau = 0$, where $N = 1000, C = 0.7$, $\langle k \rangle = 50$, and the up panels are for the network-A and down panels for the network-B. (a) and (d) represent the values of g_1 in the parameter plane of λ_{in} and ℓ_{out} for fixed $\lambda_{out} = 0.5$ and $\alpha = \pi/2 - 0.1$, (b) and (e) the case in the parameter plane of λ_{in} and λ_{out} for fixed $\ell_{out} = 500$ and $\alpha = \pi/2 - 0.1$, and (c) and (f) the case in the parameter plane of λ_{out} and α for fixed $\ell_{out} = 500$ and $\lambda_{in} = 0.8$.

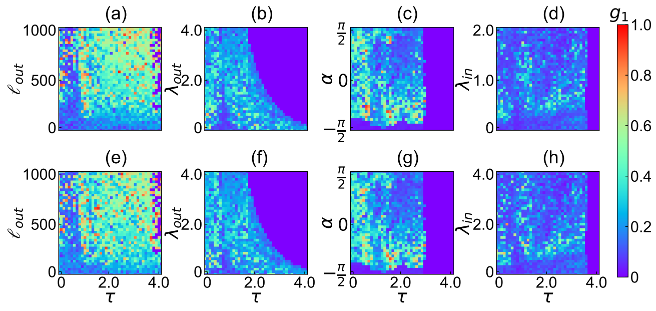

FIG. 5: (color online). Phase diagram of g_1 for the general two-layered brain network model with chemical inter-coupling of $\tau > 0$, where $N = 1000, C = 0.7$, $\langle k \rangle = 50$, and the up panels are for the network-A and down panels for the network-B. (a) and (e) represent the values of g_1 in the parameter plane of τ and ℓ_{out} for fixed $\lambda_{in} = 0.1$, $\lambda_{out} = 0.5$ and $\alpha = \pi/2 - 0.1$, (b) and (f) the case in the parameter plane of τ and λ_{out} for fixed $\lambda_{in} = 0.5$, $\ell_{out} = 500$ and $\alpha = \pi/2 - 0.1$, (c) and (g) the case in the parameter plane of τ and α for fixed $\lambda_{in} = 0.8$, $\lambda_{out} = 1.0$ and $\ell_{out} = 500$, (d) and (h) the case in the parameter plane of τ and λ_{in} for fixed $\lambda_{out} = 0.5$, $\ell_{out} = 500$ and $\alpha = \pi/2 - 0.1$.

to how τ influences the emergence of chimera state. As the revealed robustness to size N, we here only consider the case of $\tau > 0$ with $N = 1000, C = 0.7$ and $\langle k \rangle = 50$. Fig. 5 shows the results, where the individual panels represent the matching between τ and the parameters $\ell_{out}, \lambda_{out}, \alpha$ and λ_{in}, respectively. Comparing the corresponding panels between Fig. 5 and Fig. 2, respectively, we find that they are similar to each other, confirming again the equivalence between the general two-layered brain network model and the real network of cerebral cortex.

In sum, the chemical inter-coupling is helpful for the emergence of chimera state, no matter it is with or without time-delay.

References

[1] Xu, K., Zhang, X., Wang, C. & Liu, Z. A simplified memory network model based on pattern formations. *Sci. Rep.* **4**, 7568 (2014).

[2] Kopelowitz, E., Abeles, M., Cohen, D. & Kanter, I. Sensitivity of global network dynamics to local parameters versus motif structure in a cortexlike neuronal model. *Phys. Rev. E* **85**, 051902 (2012).

[3] Miledi, R. Miniature synaptic potentials in squid nerve cells. *Nature* **212**, 1240 (1966).

[4] Miledi, R. Spontaneous synaptic potentials and quantal release of transmitter in the stellate ganglion of the squid. *J. Physiol.* **192**, 379 (1967).

[5] Schutter, E. D. Computational Modeling Methods for Neuroscientists (*MIT Press, Cambridge, 2010*).
